# Supplementary figures and images for: The effect of repeat feeding on dengue virus transmission potential in Wolbachia-infected Aedes aegypti following extended egg quiescence
Source: PLoS Negl Trop Dis. 2024 Jul 8;18(7):e0012305. doi: 10.1371/journal.pntd.0012305 (PMC11257391; doi:10.1371/journal.pntd.0012305)

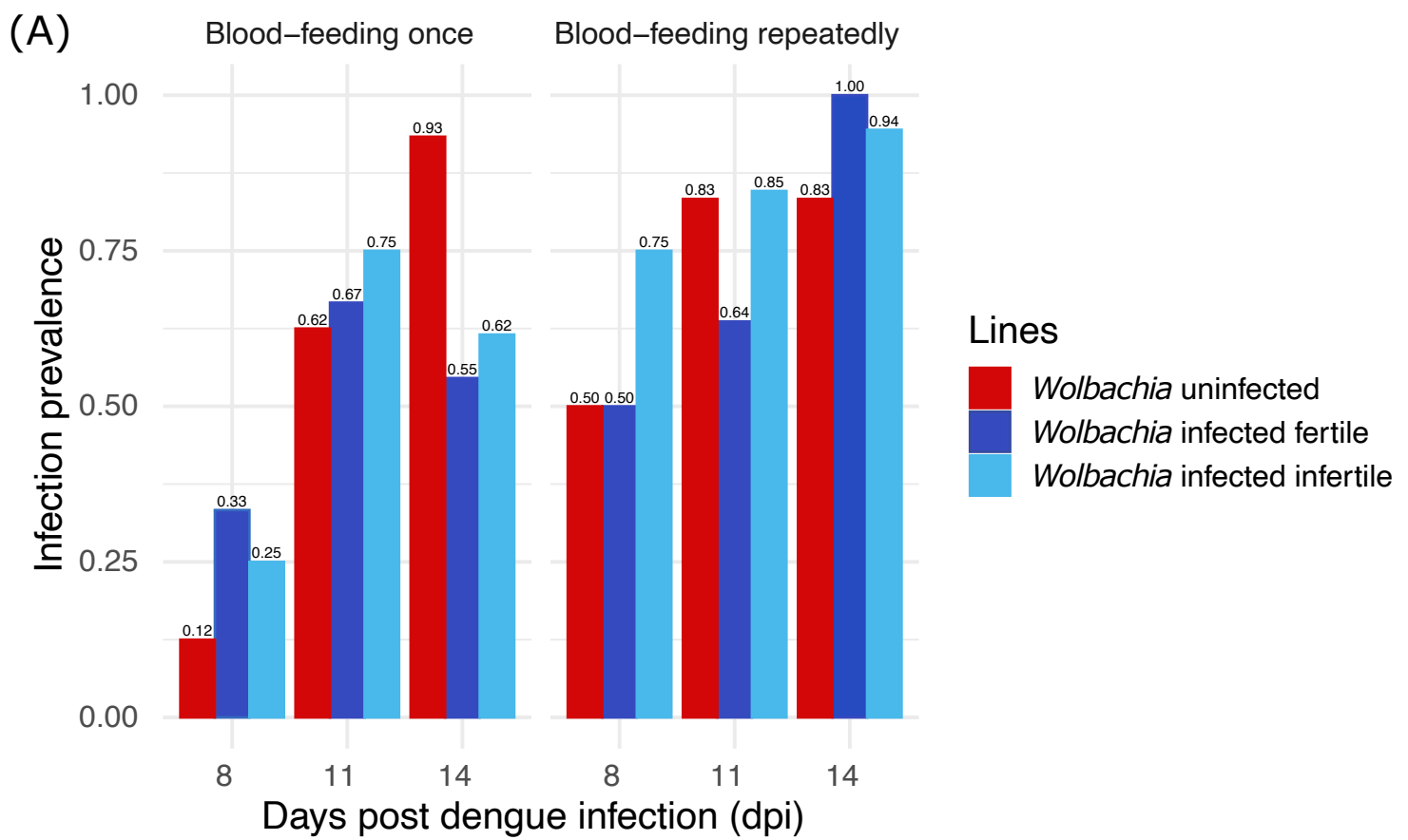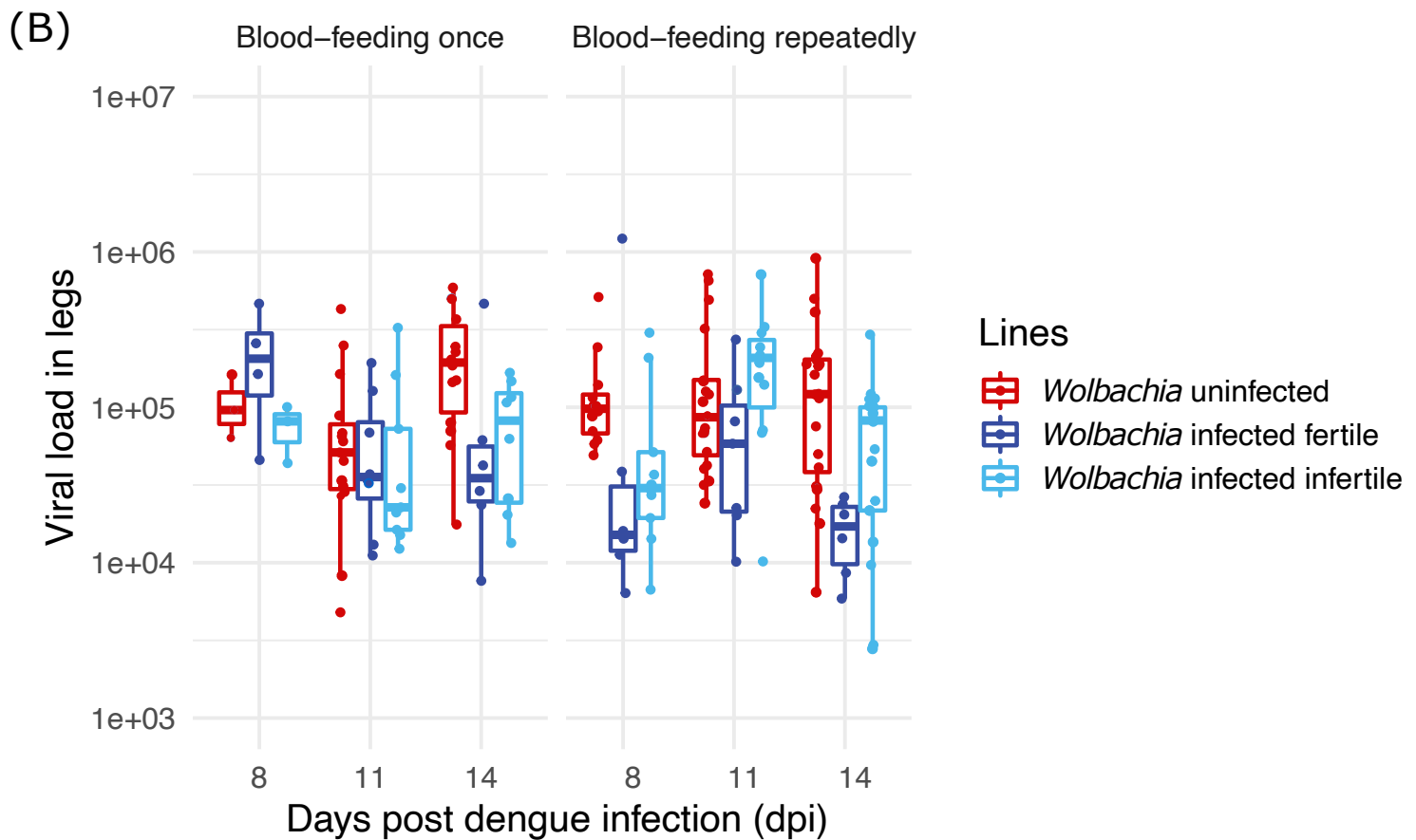

Supplement: S1 Fig — (A) DENV-2 infection prevalence and (B) DENV-2 load in adult female Aedes aegypti legs at 8, 11, and 14 days post-infection (dpi). Females were provided with infectious blood every day from 1 to 7 dpi (post the initial infectious blood meal at 0 dpi). Each collection point x treatment is represented by 24 individuals. The fertility status of Wolbachia-infected females was identified through ovarian dissection. (PDF) [file pntd.0012305.s001.pdf]

*Wolbachia* density

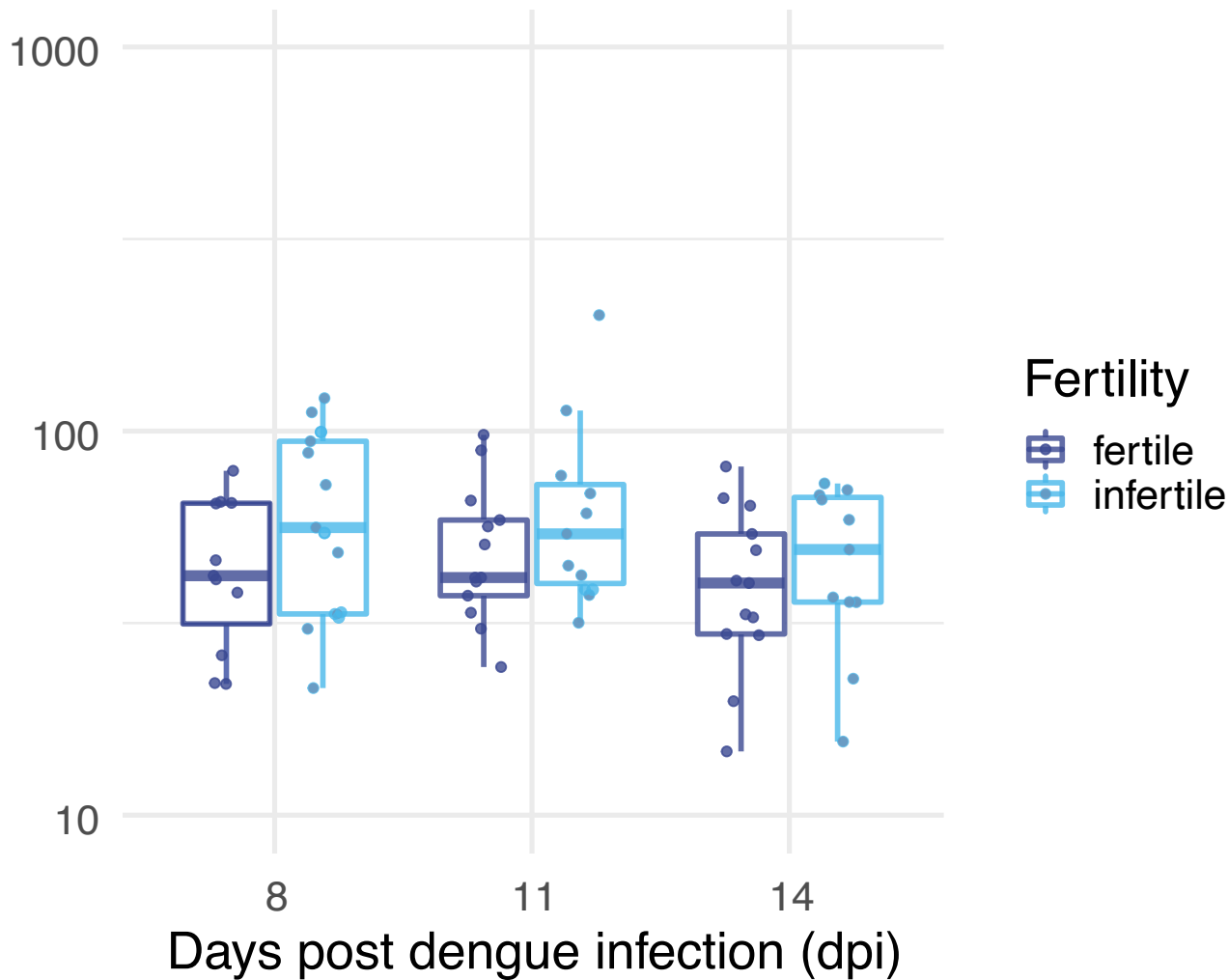

Supplement: S2 Fig — (PDF) [file pntd.0012305.s002.pdf]
