# Supplementary material for: The effect of repeat feeding on dengue virus transmission potential in Wolbachia-infected Aedes aegypti following extended egg quiescence
Source: PLoS Negl Trop Dis. 2024 Jul 8;18(7):e0012305. doi: 10.1371/journal.pntd.0012305 (PMC11257391; doi:10.1371/journal.pntd.0012305)
Supplement: Attachment — Submitted filename: MLauResponsetoReviewersMay8.docx [file pntd.0012305.s003.docx]

Dear Editor,

Please find our responses below. We are grateful for the reviewers’ comments as they have significantly strengthened the manuscript.

We grouped the integrated the queries from the different reviewers, particularly when they raised similar concerns.

**Methods**

The authors have used only samples obtained from quiescent eggs, so they cannot conclude that quiescence is a cause for those observed results. Reviewer #2: I do not agree with the first sentence in the conclusion section. Legs or other tissues such as head and midgut are fine to detect infection/prevalence rate. Salivary glands are normally used for estimating transmission rate, which is not the purpose of this study. Most of the conclusions are based on the failure of the methodological design, which seems to be the main weakness of the study. At the end, I think that the big question which is stated in the title could not be confirmed in this study, regarding the egg quiescence.
**Response.** The scale of the design was substantial with respect to all the downstream feeding and so we could not double the size with also having unstored eggs (+/- *Wolbachia*). Quiescence has already been well-established in two previous papers resulting from storage for this specific *Wolbachia* *Ae. aegypti* line. We also confirmed the effects of quiescence here – with infertility measures. We therefore focused our experimental effort on the effects of repeat feeding and the interaction with *Wolbachia*. See next paragraph about salivary glands vs. legs. We agree with both reviewers concerns about how we are describing out findings. We have as a result altered the title, intro, and discussion in response to make sure we are not overstating the meaning of the findings.

Authors adopted an experimental design in which they decided to screen DENV-2 in mosquito legs rather than in mosquito saliva. Although they recognize saliva as the golden standard for transmission dynamics, it is still unclear the reason why this design was chosen.

Was there any other tissue analyzed during the three dpi (8, 11 and 14), or were only the legs investigated? If only legs were analyzed, please replace Mosquito tissue samples with Mosquito legs.

**Response.** Our goal was to study transmission potential. This is frequently carried out by examining salivary glands or saliva. *We used legs as a proxy for transmission potential given a recent study demonstrating that it was highly effective (more effective than saliva infection) when tested with an animal model*. *Very few studies have actually tested the efficacy of the different tissues in an animal model of transmission.* Also, given the scale of the design, legs were safer (frozen mossies). Last, legs are also frequently used as to study Wolbachia mediated blocking in disseminated tissues (see references in discussion). We have been more explicit about our choice of design. We have also changed ‘tissue’ to ‘legs’ as requested.

Is there any reference about the mosquito colony used as control? If yes, please cite it in the mosquito-rearing section.

**Response.** There is no specific reference as these were collected by a colleague in Mexico several years previously and obtained from Zhiyong Xi. The line has been used for other published studies in the Xi lab, but those papers do not contain any additional information about the line compared to what we have provided. We could cite a recent paper describing the preparation for field releases for Wolbachia in Merida, but these are more recent and also do not talk about the original collections of the mosquitoes.

Both in the methodology section and in the results, they describe that they evaluated the fertility of mosquitoes with and without *Wolbachia* to compare. Therefore, it is important to make the purpose of this analysis clearer.

By stating ‘to check their fertility status by examining their ovaries to obtain the proportion of infertile females’ it would be didactic to add a reference with a picture or adding your own picture as a supplementary file.

**Response.** Good advice. We have now referenced the previous work indicating that *Wolbachia* infected females post storage in eggs frequently have undeveloped ovaries. We point readers to images in that study. We also explicitly state that we did this analysis to confirm that indeed our *Wolbachia* infected lines have high rates of infertility compared to the *Wolbachia* free.

Explain why only for the Wolbachia-infected mosquitoes the bodies were also collected.

**Response.** Legs were used for RNA extraction and virus quantification as explained above. Blocking occurs in the carcass (midgut, fat body, etc) with effects on disseminated tissues – so we wanted to use the body for Wolbachia loads. Now referenced. We only used bodies from the first feed to assess load of *Wolbachia*. *Bodies from the subsequent feeds were used to determine fertility status via dissection and were therefore not used for viral loads.*

In this section, the total number of groups investigated is not described properly. We think about four groups (1: Wolbachia infected fed once, 2: Wolbachia infected fed multiple times, 3: Wolbachia free fed once, and 4: Wolbachia free fed multiple times, however, when we see Figure 3 we observe six groups, so I suggest to explain this better in the methodology section.

**Response.** Agreed. Revised.

Please add dpi (days post infection) somewhere in methodology

**Response.** Agreed. Revised.

It was not clear to me the reason why eggs were storage for 11 weeks, not less, not more. Such research question would be better answered if a range of storage times are explored, not only a single timepoint at 11 weeks.

**Response.** Good advice. This is based on a previous study demonstrating peak infertility after 11 weeks. We have now indicated as such in the methods.

**Results**

Reviewer #2: I think that results should be described better according to groups described in the methodology sections. For instance, in the introduction the authors mention "two data sets", but it is not clear what they are. Groups are not well defined and thus, results are very confusing.
**Response.** Agreed. We have restated what we mean by this in the intro and also better defined the groups being studied across the methods and results as above.

**Discussion**

In the first paragraph the authors mention "It was recently discovered Ae. aegyp- females infected with the Wolbachia wAlbB strain can lack matured ovaries, become inferOle, and show higher feeding rates than ferOle Wolbachiainfected or wildtype uninfected females aYer experiencing an extended period of egg diapause
[3, 4]" However these two references have no information about that. Please, be aware that Aedes aegypti eggs do not enter diapause, but quiescence. Correcte that sentence and cite proper references.

**Response.** Agreed. Corrected.

The last sentence in the first page of discussion section "wolbachia-infected infertile over infertile mosquitoes" is not clear.

**Response.** Agreed. Corrected

The sentence "Our DENV prevalence data were not as expected because they lacked evidence of Wolbachia mediatedvirus blocking. In one replicate, the wild type line exhibited the lowest rates of infection and in another replicate, it did not differ from either of the Wolbachia-infected lines....We propose two explanations for the prevalence data. The first is that the lack of blocking is the result of our choice of tissue (legs)." sounds very deterministic as if it will be necessary to change the methods to find the results the authors want to find.

**Response.** Agreed. Statement removed and text revised.

The Wolbachia-infected mosquitoes probe at twice the rate of the Wolbachia-uninfected mosquitoes, even after 4 dpi. But it also reflected on a higher amount of ingested blood?

**Response.** Agreed. That is our point. We have been more explicit so it is not confusing.

The authors could mention the inconvenience of increased bites from wolbachia-infected mosquitoes released into the field, which could compromise communities' acceptance of these types of strategies.

**Response**. Great idea. Added.

**Minor comments – merged from reviewers**

Page 10 > life history traits: how was fertility status determined by examination of the ovaries? This becomes clearer later in the manuscript, but I suggest adding it to the methods section, where it first appears. **Done.**
Page 11: in most places where “media” is mentioned, it should be “medium”. For example “RPMI 1640 media”. **Done**
Pages 12 and 13, top: q-PCR > qPCR. **Done**
Page 13 > statistical analysis: Gaussian distribution **Done**
Page 15, para 2: join the two sentences “When we provided ……, infertile females….” **Done**
Page 15, last para, line 2: is Fig. 4 meant to be Fig. 3? **Yes, Done.**
1.      Methods. Traditional backcrossing would expect mating Wolbachia-infected females with wild uninfected males for around 5-6 generations. By doing so, we should expect around 80% of Mexican genetics rather than a >95%. **Backcrossing was carried out for 3 generations. We think at over 80% similar this is not likely a huge concern, regardless, we have added it to the caveats in the discussion.**
4.      Methods, Life history traits subsection, 2nd paragraph. Please add that this blood meal was composed of DENV-uninfected blood. **Done.**
5.      Methods. How to record all probing events in a cage of 30-40 individuals? Count those blood fed is ok but counting the number of probes in 30-40 starved individuals simultaneously is hard. Can you provide more details on how this data was obtained, e.g., how often it was needed to go back to video record? **Yes, it is hard! Some were watched in real time. Any that could not be were watched later by video.**
6.      Methods. Please provide more information about the DENV-2 isolate used. It seems it was isolated in Australia, but mosquitoes have a 80% Mexican background. Do authors see it as a potential source of complexity to analyze their results? **As per our data, these mosquitoes are highly competent for this virus strain.** **We did not have poor infection. The scale of this experimental design was very large already. Studying how virus variation or virus:vector genetic pairings may matter is for the future.**
7.      Results. Something is missing in the link between this sentence ‘When we provided pairwise comparisons for Wolbachia-infected fertile and infertile females’ with the others. **Fixed.**
8.      Please standardize the use of dpi or DPI. **Done.**
10.     Results. In Figure 3A, y-axis, please specify it is DENV-2 infection prevalence. **In the legend.**

Intro: First sentence should be corrected Wolbachia is widespread in arthropods not only in insects. The way it is written seems to be specific to insects. **Done.**
Some references were misused e.g. Reference 2 in the introduction section does not refers to Zika. Reference 27 is about malaria and not virus. **Fixed.**
In the second paragraph, second line, the sentence seems to lack some words ("the expression of C ,)" **Fixed.**
